# Beyond treatment: reframing child wasting prevention and recovery in humanitarian settings

**DOI:** 10.7189/jogh.16.03033

**Published:** 2026-10-23

**Authors:** Said Aden Mohamoud, Shelley Walton, Samantha Grounds, Sydney Garretson, Qundeel Khattak, Marina Tripaldi, Fabrizio Loddo, Michael Ocircan P’Rajom, Meftuh Omer Ismail, Adam Abdulkadir Mohamed, Osama Abdi Ali, Nadia Akseer

**Affiliations:** 1 Save the Children, Somalia Country Office, Mogadishu, Somalia; 2 Department of International Health, Johns Hopkins University, Baltimore, USA; 3 Save the Children, Save the Children International, London, UK; 4 Federal Government of Somalia, Ministry of Health & Human Service, Mogadishu, Somalia

**Keywords:** cash, wasting, Somalia, humanitarian, nutrition, SBCC

## Abstract

Despite substantial advances in the treatment of child wasting over the past two decades, progress in reducing wasting incidence has remained limited, particularly in humanitarian settings. This commentary argues that global policy must evolve from a predominantly treatment-oriented paradigm toward a more integrated continuum encompassing prevention, early identification, treatment, recovery, and post-discharge support. Drawing on a programme of recent research conducted in Somalia, we highlight four emerging priorities for global policy: strengthening prevention through integrated cash-plus and multisectoral approaches; improving identification of high-risk children by addressing limitations of current anthropometric indicators; recognising relapse as a major programmatic outcome requiring continued support after discharge; and integrating nutrition with broader health, social protection, and humanitarian systems. As humanitarian needs increase while development financing declines, future progress against wasting will depend not only on improving treatment, but also on rethinking how prevention, diagnosis, recovery, and system integration are addressed across the continuum of care.

Over the past two decades, remarkable progress has been made in the treatment of child wasting. The scale-up of Community-Based Management of Acute Malnutrition, simplified treatment protocols, and improved therapeutic foods have transformed wasting from a frequently fatal condition into one that can often be effectively managed in community settings [1,2]. Yet despite these advances, global progress in reducing wasting incidence has remained slow, particularly in countries affected by protracted crises [3]. This disconnect raises an important question: ‘Has the global nutrition community become too focused on treating wasting and insufficiently focused on preventing it?’

Historically, wasting has largely been conceptualised as an acute condition requiring rapid identification and treatment [2,4]. This framing has shaped both programme investments and research priorities. Consequently, much of the evidence base has focused on treatment effectiveness, protocol optimisation, and programme coverage. Comparatively less attention has been devoted to understanding pathways to prevention, the persistence of vulnerability following treatment, and the adequacy of existing diagnostic approaches for identifying children most at risk [4–12]. Emerging evidence increasingly suggests that these questions may be equally important for achieving sustained reductions in wasting and its consequences [4]. More importantly, prevention actions could be delivered at approximately USD 1.80 per child/woman per month, while early detection and treatment could cost approximately USD 90 per child admitted [13].

The challenge is particularly acute in humanitarian settings. Unlike stable contexts where improvements in economic growth, food systems, health services, and sanitation can gradually reduce undernutrition, populations affected by conflict, displacement, climate shocks, and chronic poverty face repeated and overlapping risks [3,14]. In these environments, wasting reflects the interaction of immediate causes such as inadequate dietary intake and disease, underlying causes including food insecurity, inadequate care practices, poor water and sanitation conditions, and weak health services, and broader structural determinants such as poverty, conflict, and social vulnerability [1,4,14,15]. Addressing only one of these pathways is therefore unlikely to produce sustained nutritional improvements.

This reality may help explain why evidence surrounding cash transfers and child nutrition has remained inconsistent. Across low- and middle-income countries, cash transfers have repeatedly demonstrated positive impacts on household consumption, food security, and poverty reduction [16–18]. However, effects on child anthropometric outcomes have generally been more modest and heterogeneous [16–18]. Systematic reviews have found that while cash can improve access to food, it does not automatically translate into improvements in child feeding, healthcare-seeking behaviour, hygiene practices, or intra-household resource allocation [16,17]. Ultimately, nutrition outcomes are shaped by a combination of resources, knowledge, agency, and environmental conditions [4,15].

Somalia provides a particularly important setting for examining these questions, given the convergence of multiple factors that increasingly shape child malnutrition globally, including protracted conflict, recurrent climate shocks, large-scale displacement, chronic food insecurity, and constrained health-system capacity [19–21]. While much of the evidence informing global wasting policy has historically been generated in relatively stable settings or has focused on treatment outcomes, there remains limited evidence on how wasting can be prevented, accurately identified, and sustainably addressed in humanitarian contexts. The other studies referenced here [22–26] were designed to address several of these critical evidence gaps by examining the effectiveness of integrated cash-plus interventions, the determinants of wasting, the performance of alternative anthropometric indicators, and the burden of post-treatment relapse. Collectively, these questions reflect a broader shift occurring within global nutrition discourse, from a narrow focus on treatment and programme recovery rates toward a more holistic understanding of wasting as a dynamic condition shaped by interconnected biological, social, economic, and environmental factors. By generating rigorous evidence in one of the world’s most challenging humanitarian settings, these studies contribute not only to policy and programming in Somalia, but also to ongoing global efforts to rethink how wasting prevention, diagnosis, treatment, and recovery can be integrated across the continuum of care.

The Somalia evidence [22–26] contributes to this growing literature by suggesting that the nutritional effectiveness of cash may depend less on the transfer itself and more on the extent to which transfers are embedded within broader systems of support, while acknowledging that transfer size, timing and contextual shocks are also key influencing factors. Findings from the adaptive cluster-randomised trial [22] indicate that cash alone was insufficient to reduce wasting, whereas cash combined with structured social and behaviour change communication generated measurable reductions in wasting and represented a cost-effective strategy [22]. These findings are consistent with a growing body of evidence suggesting that integrated approaches that simultaneously address economic, behavioural, and social determinants may be more effective than interventions targeting a single constraint in isolation [4,15,19]. Importantly, qualitative findings from the Somalia studies [22] indicate that caregivers valued practical support through mother-to-mother support groups and cooking demonstrations, suggesting that behavioural and social components may play a critical role in translating financial resources into improved child nutrition outcomes.

Importantly, these findings arrive at a time when humanitarian systems are undergoing profound transformation. Major reductions in development and humanitarian financing are forcing governments and agencies to make increasingly difficult choices regarding programme prioritisation [27]. Official development assistance decreased by 23.1% overall in 2025, with declines in USA government funding alone decreasing by about 57%, driving three-quarters of the total decline [28]. Under such circumstances, the question is no longer simply whether interventions work, but whether they generate sufficient impact to justify increasingly scarce resources. The Somalia findings suggest that integrated approaches could represent one pathway toward improving efficiency, yet they also expose persistent structural barriers. In Somalia, nutrition, health, food security, and cash assistance programmes often operate through separate delivery systems, financing streams, and coordination mechanisms, limiting opportunities for synergy and scale [20].

A second lesson emerging from the Somalia experience concerns the continued uncertainty surrounding how acute malnutrition should be measured. For decades, debates regarding the relative merits of weight-for-height z-scores (WHZ) and mid-upper arm circumference (MUAC) have remained unresolved [5–8]. Operational considerations have often favoured MUAC because of its simplicity and feasibility in humanitarian settings [4]. However, growing evidence demonstrates that different anthropometric indicators identify substantially different populations of children [5–8]. The Somalia findings reinforce this concern, demonstrating striking discordance between MUAC and WHZ classifications [25]. These results are consistent with studies from other contexts showing that reliance on a single indicator may fail to identify large numbers of nutritionally vulnerable children [5–8].

Yet the policy implications are not straightforward. Expanding diagnostic criteria could improve case detection, but it could also dramatically increase programme caseloads in already resource-constrained settings. This tension highlights a broader challenge facing global nutrition policy: scientific evidence and operational feasibility do not always align. The question is therefore not only which indicator best predicts risk, but how programmes can responsibly implement improved diagnostic approaches while maintaining quality and coverage [4].

Perhaps the most neglected issue identified in these studies is relapse into wasting. Historically, programme success has been measured primarily through recovery rates achieved during treatment. Far less attention has been given to what happens after discharge [9–12]. This omission reflects an implicit assumption that anthropometric recovery signals resolution of the underlying problem. Increasing evidence suggests otherwise. Studies from multiple countries have documented substantial relapse following treatment, raising concerns that recovery may often represent a temporary improvement rather than sustained nutritional resilience [9–12].

The Somalia findings add important prospective evidence for this emerging literature. Nearly one-third of recovered children relapsed within six months, with the highest risk concentrated immediately after discharge [26]. These findings challenge conventional notions of programme success and suggest that discharge may represent not the end of treatment, but the beginning of a particularly vulnerable transition period. Viewed through this lens, wasting management should be conceptualised less as a discrete episode of care and more as a continuum encompassing prevention, treatment, recovery, and post-discharge support, consistent with the 2023 World Health Organization guidelines on the prevention and management of wasting [4].

Taken together, these findings point toward the need for a broader conceptual shift in global wasting policy. The dominant model remains heavily oriented toward identifying and treating individual episodes of acute malnutrition. While treatment remains essential, this approach risks addressing the symptoms rather than the systems that produce recurrent wasting. A more effective strategy may require integrating nutrition programming with social protection, health services, food systems interventions, and caregiver support while simultaneously strengthening efforts to prevent relapse among children who have already experienced wasting.

The Somalia studies do not provide all the answers. Important questions remain regarding scalability, sustainability, financing, and implementation in diverse humanitarian contexts. Nevertheless, they contribute to a growing body of evidence suggesting that future progress against wasting will depend not only on improving treatment, but on fundamentally rethinking how prevention, diagnosis, and recovery are approached. As humanitarian needs continue to grow while resources contract, the imperative is clear: programmes must move beyond isolated interventions and toward integrated systems capable of addressing the complex and interconnected determinants of child malnutrition.

## Data Availability

**Data availability:** These data were collected on a vulnerable population linked to a large-scale cash assistance programme in Somalia. The data may be made available upon request with permission from local study investigators.
